# Nasal cavity perforation by implant fixtures: case series with emphasis on panoramic imaging of nasal cavity extending posteriorly

**DOI:** 10.1186/s13005-023-00384-z

**Published:** 2023-08-22

**Authors:** Han-Gyeol Yeom, Kyung-Hoe Huh, Won-Jin Yi, Min-Suk Heo, Sam-Sun Lee, Soon-Chul Choi, Jo-Eun Kim

**Affiliations:** 1 Department of Oral and Maxillofacial Radiology and Wonkwang Dental Research Institute, College of Dentistry, Wonkwang University, Iksan, Korea; 2 Department of Oral and Maxillofacial Radiology and Dental Research Institute, School of Dentistry, Seoul National University, Seoul, Korea

**Keywords:** Dental implant, Nasal cavity, Panoramic radiography, Case report

## Abstract

The nasal cavity is an important landmark when considering implant insertion into the anterior region of the maxillary arch. The perforation of implants into the nasal cavity may cause complications, such as implant migration, inflammation, or changes in nasal airflow; thus, precise assessment of the nasal cavity is mandatory.

Three cases of nasal cavity perforation by dental implants are presented, including one case of implant fixture migration into the nasal cavity. On panoramic radiographs of the patients, the following common features were observed: the horizontal radiopaque line of the hard palate was observed to be inferior to or similar to that of the antral floor and the bone between the lateral wall of the nasal cavity and the medial wall of the maxillary sinus was emphasized in a triangular shape.

When the maxillary sinus is small and alveolar bone resorption is severe, panoramic evaluation may cause overestimation of the available residual bone, particularly in the maxillary canine/premolar region. Therefore, the residual bone should be reevaluated three-dimensionally to measure the exact bony shape and volume.

## Background

The development and advancement of dental implants in recent years have had a significant impact on overall dental treatment planning. In preoperative treatment planning, including the insertion of dental implants, it is essential to determine the optical position, angulation, number and size of the implants. This can be achieved by accurately determining the dimensions and shape of the jaws and the location of vital anatomical structures, especially the inferior alveolar canal in the mandible, maxillary sinuses and nasal cavity in the maxilla [1].

Panoramic radiography, intraoral radiography, and cone-beam computed tomography (CBCT) are imaging modalities commonly used in the diagnostic process of implant placement [2]. Panoramic radiograph has been widely used, as it has the advantages of being cost-effective, readily available, and it provides high-resolution images [3]. Additionally, it is possible to evaluate the overall jawbone using a single image. However, it is difficult to evaluate the ideal position and anatomical structure in the buccolingual direction on account of the limitations of two-dimensional images [1]. Furthermore, there is some degree of unavoidable distortion [4]. The focal layer of the incisor region in panoramic radiography is sometimes too narrow to provide sufficient information.

Nevertheless, in many cases, the overall bony information is still obtained through panoramic radiographic images. Panoramic radiography can be used as a final diagnostic imaging modality in the planning of dental implants when the patient’s bone anatomy is not sufficiently disadvantageous to require CBCT imaging or when it is difficult to perform CBCT in a clinical setting [5]. However, if three-dimensional structural features cannot be sufficiently determined from the acquired panoramic radiograph, three-dimensional imaging should be acquired accordingly. Therefore, clinicians should be able to accurately understand panoramic radiographs and also determine the need for additional images to be acquired from the information obtained during this procedure.

When placing implants in the maxilla, the maxillary sinus is evaluated as the most important structure for obtaining sufficient bone volume, which affects the initial fixation [6]. If necessary, bone grafting can be performed using the space within the sinus cavity to secure a minimum amount of bone. There have been many reports regarding implant migration into the maxillary sinus [7–10].

The nasal cavity is another important landmark when considering implant insertion into the anterior region of the maxillary arch. The perforation of implants into the nasal cavity may cause complications, such as implant migration, inflammation, or changes in nasal airflow; thus, precise assessment of the nasal cavity is mandatory [11, 12]. However, there have been relatively few reports on perforation of the nasal cavity reported [11–16]. Most of the reported cases were related to anterior region of the maxilla [11–15], and among them, only two cases showed complete implant fixture migration [14, 15]. In one case, the implant fixture migrated and passed from the maxillary sinus through the sinus ostium into the nasal cavity [14]. There was only one case of direct migration into the nasal cavity, which involved two fixtures [15]. However, none of these cases discussed the association with posterior dental implant perforation of the nasal cavity. Park et al. first conducted an analysis of implants accidentally penetrating the nasal cavity in the posterior maxilla, focusing on inferior nasal cavity enlargement, referred to as “inferior meatus pneumatization” [16]. According to the study, the inferior meatus pneumatization extended up to the second molar area, and the sinus floor was cranially located concerning the nasal floor in all cases in radiologic finding by the CBCT images. However, this study did not address the features observable on panoramic radiographs. To the best of our knowledge, there have been no studies that considered the association of nasal cavity perforation with significant panoramic imaging features of the nasal cavity.

Clinicians do not expect nasal floor penetration of implants in the posterior maxilla due to the infrequency of inferior nasal cavity enlargement. Moreover, nasal cavity enlargement is challenging to detect in two-dimensional radiographic views [16]. If it is possible to predict a high likelihood of nasal cavity enlargement from routinely taken panoramic radiographs and consider this in the treatment planning for dental implants, it is expected that more appropriate imaging recommendations can be provided, and suitable treatment plans can be formulated.

The purpose of this case report is to present and analyze three instances of nasal cavity perforation caused by dental implants. Additionally, we aim to discuss the significant panoramic imaging characteristics of the nasal cavity and their relationship with the adjacent maxillary sinus. Throughout this enhanced understanding of the nasal cavity and maxillary sinus areas in panoramic radiographs, we try to offer valuable insights to ensure a more comprehensive evaluation during the planning of implant treatments.

## Case presentation

Panoramic radiographs of all patients were obtained using OP-100® (Instrumentarium Dental, Tuusula, Finland) at the the Seoul National University Dental Hospital, Seoul, South Korea. The images were obtained with optimal parameters according to the user manual for imaging adult males: 73 kVp, 10 mA, and 17.6 s, which are routinely used in the department. This retrospective study was approved by the institutional review board and written informed consent was obtained from the patients for the publication of this case report and any accompanying images (IRB no. ERI21035).

### Case 1

**Fig. 1:**
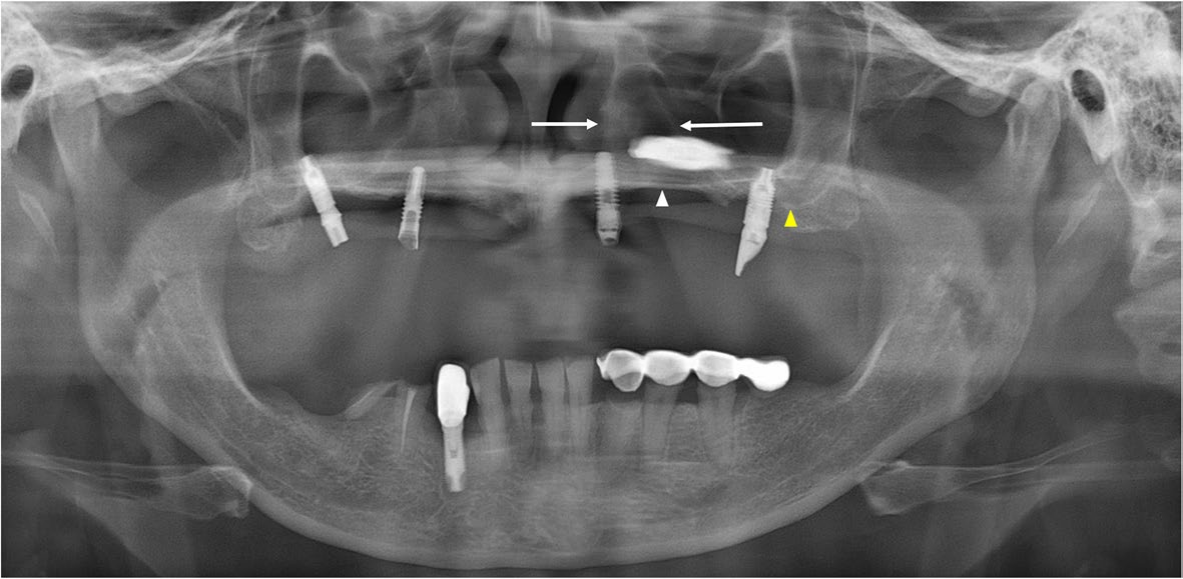
Panoramic radiograph of case 1 patient. The white arrowhead indicates the left hard palate line and the yellow arrowhead indicates the antral floor. The implant fixture is displaced into the superior cavity; however, the bone between the white arrows appears to have sufficient volume

A 51-year-old woman was referred to our hospital on account of migration of the implant fixture to the superior cavity. The panoramic radiograph revealed that one implant fixture, which was intended to be placed in the premolar region, had been displaced superiorly (Fig. 1).

**Fig. 2:**
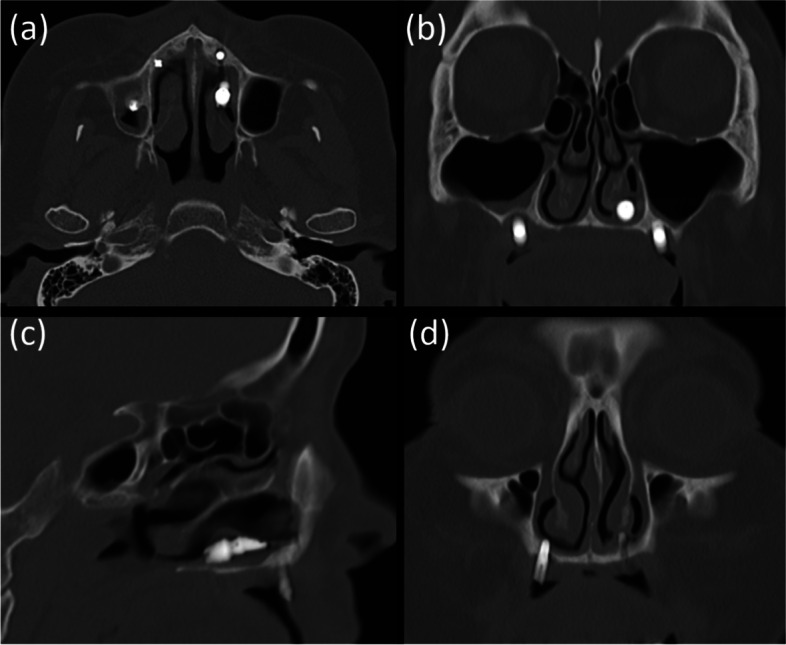
CT images of case 1 patient. The implant fixture was displaced in the left nasal cavity but not in the maxillary sinus **a**-**c**. The implant fixture of the right premolar area, which appeared to be placed in a place with insufficient bone between the nasal cavity and the maxillary sinus in panoramic radiograph, is penetrating the nasal floor **d**

The implant was observed horizontally between the boundaries of the nasal cavity and maxillary sinus, and it appeared that the fixture perforated the medial wall of the maxillary sinus. The fixture image was slightly enlarged and blurred. On computed tomography (CT) images acquired at the same time, the implant fixture was confirmed not in the maxillary sinus but in the nasal cavity (Fig. 2). It was completely different from what was predicted by the panoramic images.

In addition, on panoramic radiography, it appeared that a significant amount of bone was available between the lateral side of the nasal cavity and the medial side of the maxillary sinus. The implant fixture of the right premolar area appeared to be well placed with sufficient bone between the nasal cavity and maxillary sinus (Fig. 1), although the height of the alveolar bone was low. However, the acquired CT showed that there was insufficient bone in the area, and the implant in this area penetrated the nasal floor (Fig. 2d).

### Case 2

**Fig. 3:**
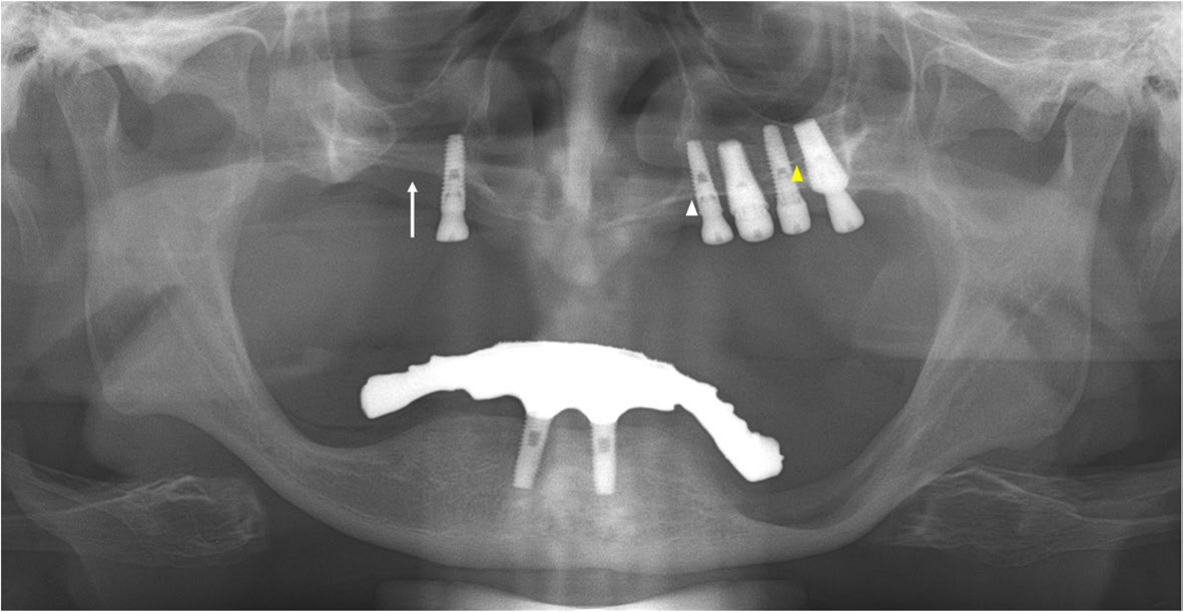
Panoramic radiograph of case 2 patient. The perforation is suspected at the right maxillary sinus floor (white arrow). The white arrowhead indicates the left hard palate line and the yellow arrowhead indicates the antral floor

A 64-year-old man was referred for an oronasal fistula. The patient complained that the implant treatment failed three times in the right maxilla. On panoramic radiography, perforation was suspected at the right maxillary sinus floor (Fig. 3), and one retained fixture in the maxillary right canine area was observed just lateral to the right nasal cavity.

**Fig. 4:**
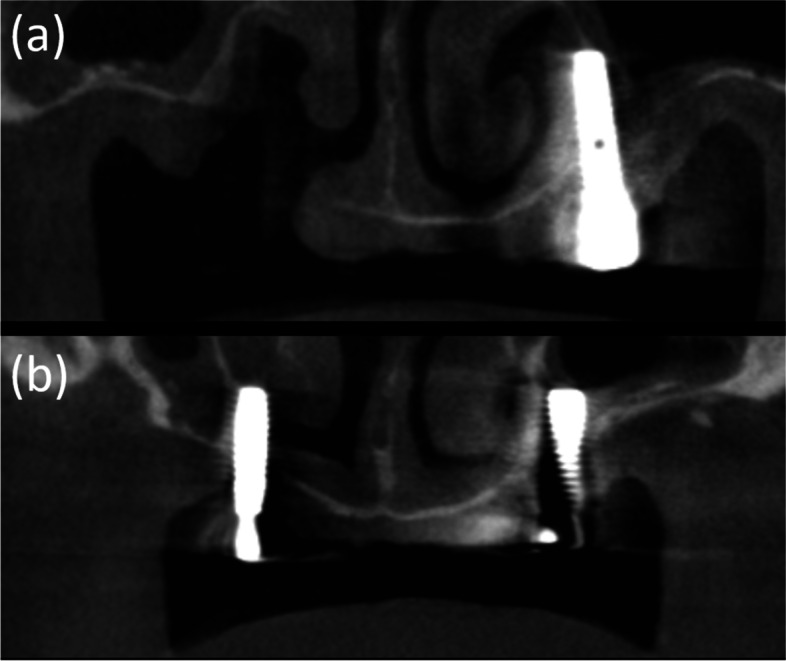
CBCT images of case 2 patient. An obvious discontinuity of the inferolateral cortex of the nasal cavity and oronasal fistula, which is observed as a tissue defect on panoramic radiography, is observed due to explantation **a**. Moreover, the residual fixtures on both sides of the maxilla perforated the nasal cavity and are placed just medial to the maxillary sinus **a**, **b**

 When CBCT was performed, obvious discontinuity of the inferolateral cortex of the nasal cavity and oronasal fistula, which was observed as a tissue defect on panoramic radiograph, was observed due to explantation (Fig. 4). Moreover, the residual fixtures on both sides of the maxilla perforated the nasal cavity and were placed just medial to the maxillary sinus.

On the panoramic radiograph, the boundaries of the lateral wall of the nasal cavity were relatively clearly observed. Resorption of the maxillary alveolar bone was severe; however, residual bone remained in the canine/premolar region, lateral to the lateral wall of the nasal cavity. In contrast, CBCT revealed that the implant fixtures of the left maxilla were narrowly placed along the boundaries of the nasal cavity and the maxillary sinus (Fig. 4a, b).

### Case 3

**Fig. 5:**
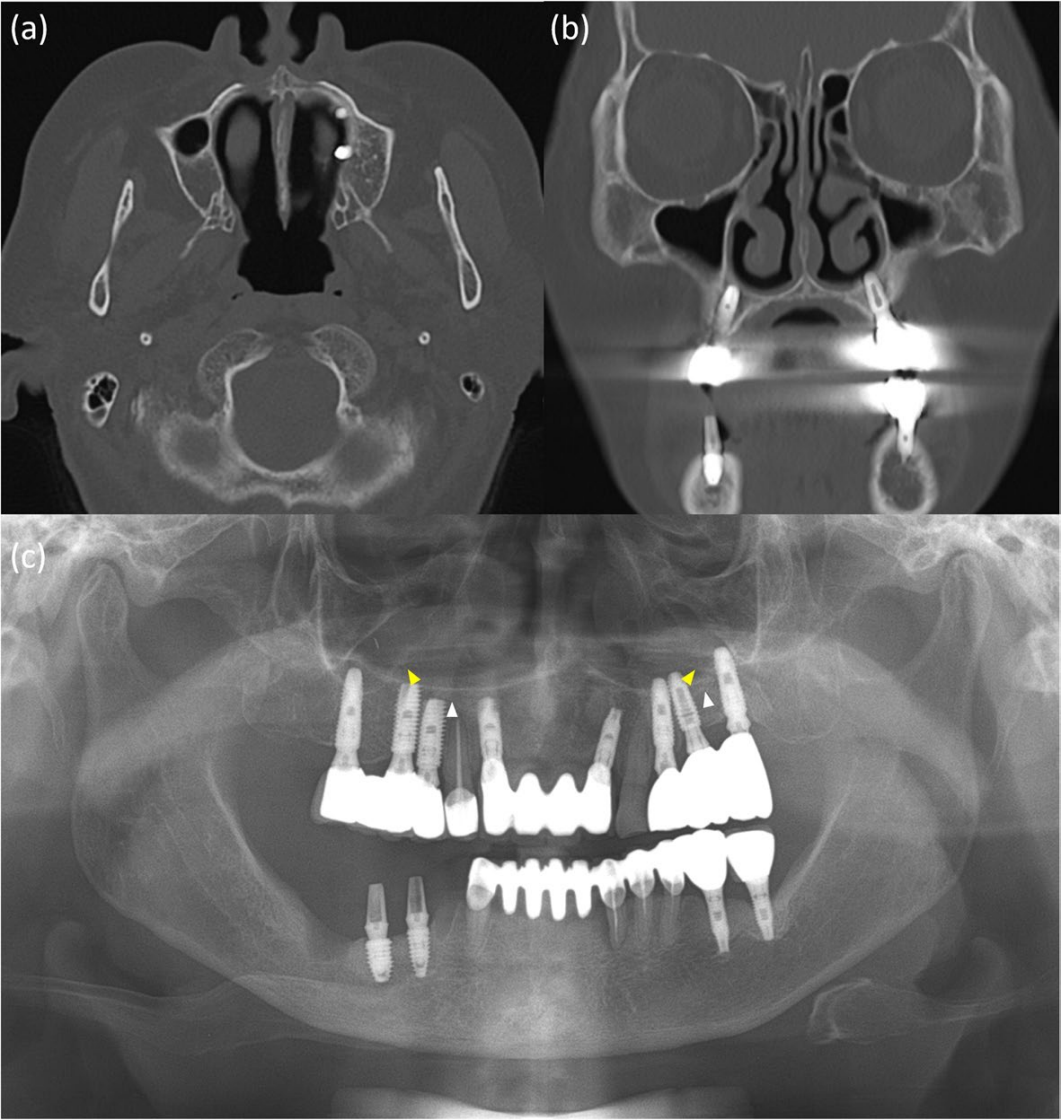
**a** Panoramic radiograph of case 3 patient. White arrowheads indicate the hard palate line and yellow arrowheads indicate the antral floor. Images of the implant fixtures in the left premolar and molar areas are superimposed above the hard palatal line. **b**, **c** CT images of case 3 patient. Nasal floor perforation by implant fixtures of maxillary left premolar is observed

A 76-year-old woman visited our clinic with complaints of pain in the mandibular right posterior region and was diagnosed with osteomyelitis of the right posterior mandible based on clinical and radiological examinations. On CT images, nasal floor perforation by implant fixtures of the maxillary left premolar was observed by chance, and the patient did not complain of any apparent discomfort in this area. However, panoramic radiographs showed that the fixtures of the left maxillary premolar region were placed normally between the lateral wall of the nasal cavity and the faint medial wall of the maxillary sinus (Fig. 5). The lateral wall of the nasal cavity and the mesial wall of the maxillary sinus showed a faint border, however the radiopaque line of the hard palate extending from the nasal floor was obvious on panoramic radiography. In this case, it was shown that with the perforation containing only mucosa in a small area, the implant could be maintained well without side effects, such as the patient’s distinct symptoms or airway changes.

## Discussion

The insertion of dental implants into the maxillary sinus is the most common complication that occurs in the maxilla, and various studies have been conducted on the shape of the maxillary sinus associated with implant placement. In addition, various studies have been conducted on the factors affecting the size and shape of the maxillary sinus and their influence on panoramic features [6, 17, 18].

The penetration of dental implants into the nasal cavity is another complication. Despite the fact that it can be asymptomatic and may remain in the nose for many years [16, 19, 20], when complications do occur, unilateral mucopurulent and nasal discharge are the most prevalent symptoms in such cases [11]. It can also alter airflow accompanied by pain and discomfort [16, 21]. Hence, it is important to evaluate the nasal cavity by radiological examination to avoid perforation and secure an appropriate bony volume. Also, if the alveolar bone is narrow, implant placement requires increased attention, and a more meticulous assessment is necessary even after the placement.

This study showed three cases of implant penetration in the nasal cavity with varying severities. The common feature of the (CB)CT images of the presented patients was that the size of the maxillary sinus on the affected side was relatively small and the nasal cavity was relatively widened horizontally. In a study by Park et al., the three-dimensional CBCT image was analyzed using the concept of inferior meatus pneumatization as a characteristic of the presence of implant fixtures invading the enlarged nasal cavity [16]. This was similar to the presented three cases; CBCT showed relatively small maxillary sinus and enlarged inferior meatus. We focused on how these changes appear in the panoramic radiographs and found the following common features: (1) the horizontal radiopaque line of the hard palate was observed to be inferior to or similar to that of the antral floor; (2) the bone between the lateral wall of the nasal cavity and the medial wall of the maxillary sinus was emphasized and observed in a triangular shape, as if the bony volume was larger than the actual three-dimensional volume.

Understanding the image of the nasal cavity in a panoramic radiograph cannot be explained simply. The overall changes in the volume and shape of the nasal cavity, adjacent maxillary sinus, and their three-dimensional relationship with the focal layer on panoramic radiography have a complex effect on the obtained image features of the nasal cavity.

**Fig. 6:**
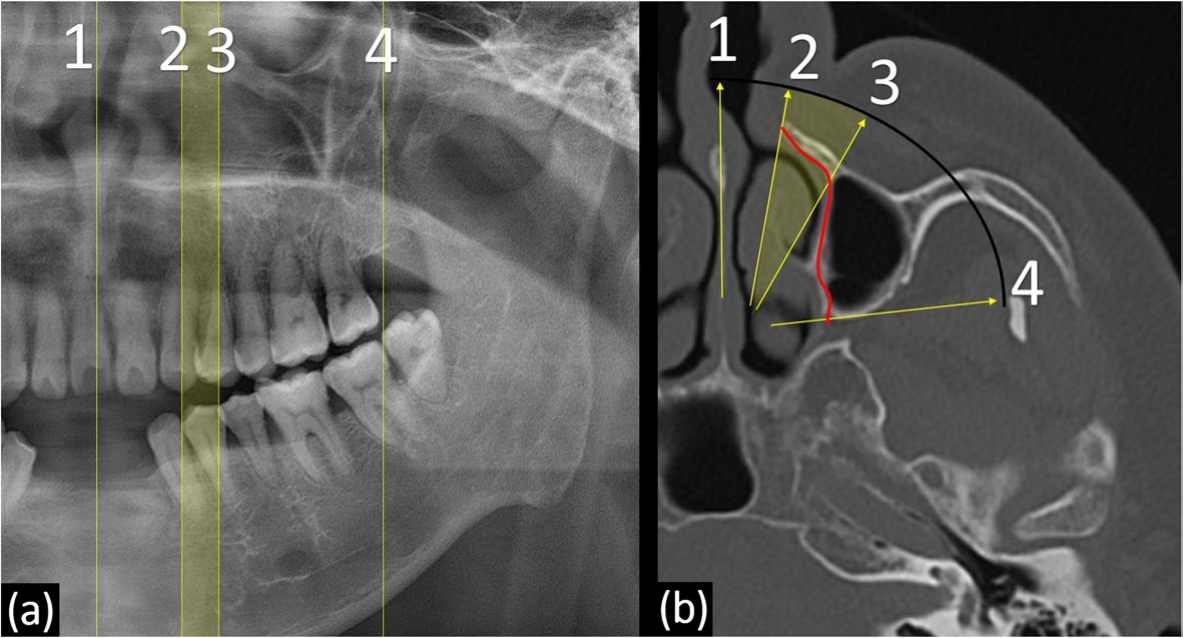
The movement and process of the central ray of the panoramic radiograph around nasal cavity is matched and marked by same number. The vertical lines of the panoramic radiography (**a**) and the yellow arrows of the three-dimensional image (**b**) shows the path through which the central ray of the X-ray beam passes at the moment. When a central ray passes through the nasal septa, it passes vertically (number 1). As the central ray moves, it passes the corner of the abduction side, the nasal pyriform aperture of the maxilla (number 2), rather than the actual lateral wall (red line)

 The lateral demarcation line of the nasal cavity in the panoramic image is the nasal pyriform aperture rather than the actual lateral wall of the nasal cavity in the actual three-dimensional anatomy (Fig. 6, line 2).

**Fig. 7:**
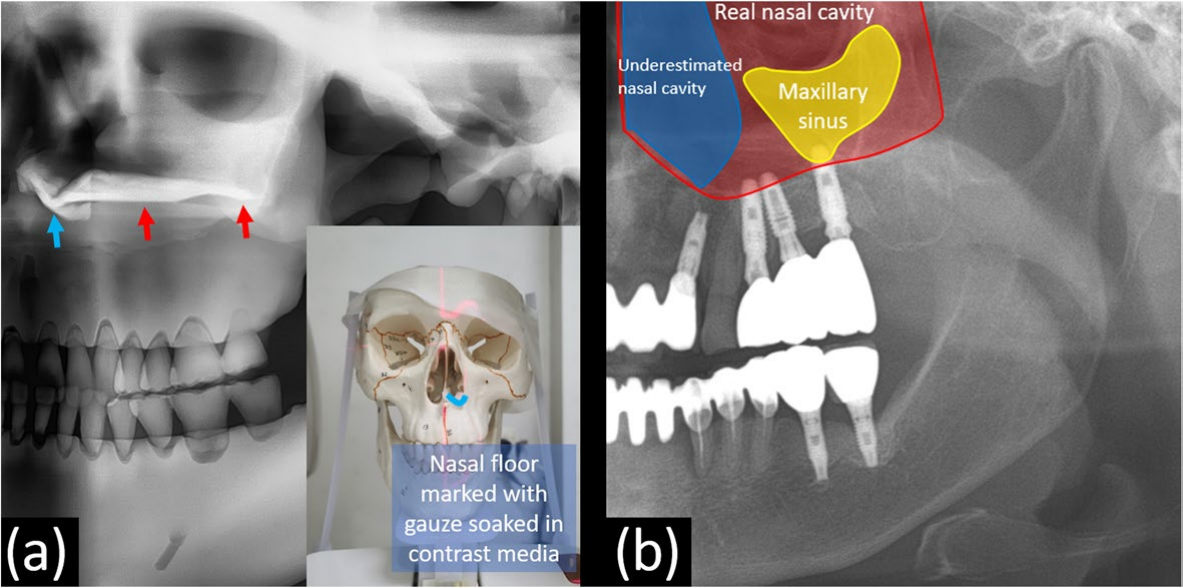
**a** In order to confirm the panoramic features of the nasal floor, the gauze soaked in contrast media is placed on the nasal floor of the plastic skull and a panoramic radiograph is obtained. The three-dimensional nasal floor not only forms a radiopaque line below the demarcated nasal cavity (blue arrow) but it also extends posteriorly as a radiopaque line, often known as the hard palate line, which is anatomically the palatal process of the maxilla (red arrow). **b** The actual nasal cavity is present in the panoramic image, with a long posterior overlap along the nasal floor line

 In addition, the three-dimensional nasal floor not only forms the radiopaque line below the demarcated nasal cavity mentioned above but it also extends posteriorly as a radiopaque line, often known as the hard palate line, which is anatomically the palatal process of the maxilla (Fig. 7a) [22]. This phenomenon is similar to the posterior extension of the inferior nasal concha observed on panoramic radiography. The nasal cavity and maxillary sinus are three-dimensionally separated adjacent structures; however, in obtaining panoramic radiographs, both the nasal cavity and maxillary sinus exist in the same path through which X-rays pass. Even though the actual nasal cavity is present with a long posterior overlap along the nasal floor line (Fig. 7b), it can be mistakenly assumed that only the maxillary sinus, with its boundaries respectively visible, provides information in that region in the panoramic image. For example, in the case 1, when clinical dentists first encounter the panoramic radiograph, there is a high probability of erroneously assuming that implants have been placed in the maxillary sinus, as implant fixtures can be seen on the outer side of the corticated nasal cavity boundary, leading to misinterpretation. Therefore, when interpreting such radiographs, it is crucial not to overlook the fact that information from both the maxillary sinus and the nasal cavity is presented together. Especially when the maxillary sinus appears small, considering that the nasal cavity occupies a broader area in that region can aid in the accurate interpretation.

In cases of hypoplasia or atrophy of the maxillary sinus, three-dimensional reduction of the maxillary sinus displaces the anteromedial margin of the sinus posteriorly and the antral floor superiorly. The greater the anterior-posterior difference between numbers 2 and 3 (Fig. 6b), the larger the area between numbers 2 and 3 in the panoramic image (Fig. 6a), which causes an overestimation of the available maxillary bone in panoramic radiographs. In addition, the antral floor line is observed to be similar or superior to the hard palatal line.

Yoshida et al. revealed the influence of depression of the maxillary sinus anterior wall on panoramic radiographic appearance [17]. They showed that the diagonal line on a panoramic image was related to the depression of the anterior wall of the maxillary sinus. Since sinus volume decreased due to inward retraction of the maxillary sinus walls, including not only the anterior wall but also the medial or inferior wall, this phenomenon can be closely related to the imaging features presented in this paper. Therefore, in the panoramic features, the lateral region of the reduced maxillary sinus could be related to the diagonal line, the medial wall could be related to the triangular overestimated bone, and the floor could be related to the similar heights of the nasal and antral floors.

Normally, even if this phenomenon occurs, it does not cause problems. However, if alveolar bone resorption is severe and implant placement is considered in the maxilla, panoramic evaluation may cause overestimation of the available residual bone, particularly in the maxillary canine/premolar region. In addition, since the alveolar bone of the maxilla is in buccoversion, bone resorption causes an unfavorable bone shape in which implantation can be planned towards the nasal cavity [23]. This can influence the treatment plan. If the superior region of planned implant location is not the maxillary sinus, it may lead to changes in the plan regarding bone grafting and other considerations. Moreover, there could be possibilities of changing the implant location to a more favorable position. Therefore, if panoramic radiographs reveal the aforementioned features, careful evaluation of the residual bones using additional three-dimensional images is required accordingly.

The clinical importance of this information lies in the ability to acknowledge the potential occurrence of nasal cavity perforation as an accidental complication during the surgical procedure. Understanding the posterior extension of the nasal cavity in panoramic imaging and being aware of its presence are crucial in reducing complications resulting from nasal cavity perforation. This knowledge significantly influences the establishment of treatment plans, including the selection of appropriate implant types and their placement. In addition, it helps to properly decide the need for additional three-dimensional imaging, which may also influence the determination of the ideal implant placement position in the treatment plan.

The limitation of this report is that only three cases were presented and discussed, and it did not address how presented common panoramic features can manifest diversely in actual patients. Also, further research through well-designed experiments is necessary to investigate how phenomena such as nasal cavity pneumatization and sinus hypoplastic change increase with age and how these changes are reflected in panoramic imaging characteristics.

## Conclusions

When the maxillary sinus is small and alveolar bone resorption is severe, panoramic evaluation may cause overestimation of the available residual bone, particularly in the maxillary canine/premolar region. Therefore, the residual bone between the lateral wall of the nasal cavity and the medial wall of the maxillary sinus should be reevaluated three-dimensionally to measure the exact bony shape and volume.

It should also be understood that the three-dimensional nasal floor not only forms a radiopaque line below the nasal cavity demarcated in the panoramic radiograph but it also extends posteriorly as the inferior nasal concha does.

## Data Availability

All data analyzed in this study are included in this published article.

## Abbreviation

CBCT: Cone-beam computed tomography
CT: Computed tomography
